# Formation of crystal-like structures and branched networks from nonionic spherical micelles

**DOI:** 10.1038/srep17941

**Published:** 2015-12-09

**Authors:** Joshua J. Cardiel, Hirotoshi Furusho, Ulf Skoglund, Amy Q. Shen

**Affiliations:** 1 Micro/Bio/Nanofluidics Unit, Okinawa Institute of Science and Technology Graduate University, Okinawa, Japan; 2 Structural Cellular Biology Unit, Okinawa Institute of Science and Technology Graduate University, Okinawa, Japan

## Abstract

Crystal-like structures at nano and micron scales have promise for purification and confined reactions, and as starting points for fabricating highly ordered crystals for protein engineering and drug discovery applications. However, developing controlled crystallization techniques from batch processes remain challenging. We show that neutrally charged nanoscale spherical micelles from biocompatible nonionic surfactant solutions can evolve into nano- and micro-sized branched networks and crystal-like structures. This occurs under simple combinations of temperature and flow conditions. Our findings not only suggest new opportunities for developing controlled universal crystallization and encapsulation procedures that are sensitive to ionic environments and high temperatures, but also open up new pathways for accelerating drug discovery processes, which are of tremendous interest to pharmaceutical and biotechnological industries.

Surfactants are amphiphillic molecules that consist of a bulk hydrophilic head group, which can be neutrally, positively, or negatively charged, and a hydrophobic tail group^1^. Surfactant molecules can self-assemble into different morphologies such as spherical micelles, cylindrical micelles, vesicles, and lamellar phases, which have found applications in consumer and pharmaceutical products, nanotemplating and biomedical applications^2^,^3^,^4^,^5^,^6^,^7^. The morphology of these self-assembled structures is directly related to the concentration of the surfactant, temperature, external additives, and flow conditions^1^,^2^,^3^,^4^. Creating higher order nano- and micro-structures from nonionic surfactants is desirable due to their electrolyte-free environment, biocompatibility, and less eco-toxic properties^5^,^6^,^7^,^8^,^9^,^10^. One exciting area of research is to use nonionic surfactant solutions as potential precursors to create branching and crystal-like structures for encapsulation, purification, and crystallization applications^11^,^12^.

The transition from nonionic spherical micelles into wormlike micelles and branched micelles has been typically achieved by increasing temperatures or/and imposing external flows^10^,^13^,^14^,^15^,^16^,^17^,^18^,^19^,^20^,^21^,^22^,^23^,^24^,^25^. For example, Safran and co-workers induced nonionic wormlike to branched micelles transition by increasing temperatures at equilibrium^20^,^21^,^22^,^23^. Higher temperature can significantly affect the solubility of nonionic micelles and their headgroup packings. As a result, the spontaneous curvature of nonionic amphiphilic molecules can be effectively reduced, making it energetically less favorable to form hemospherical end-caps. Consequently, one-dimensional micellar growth is preferred at higher temperatures, ultimately leading to branched micelle formation, controlled by a complex balance among mixing entropy at molecular levels, entropy of the topological defects, and curvature energy associated with the defect geometry^10^,^13^,^20^,^21^,^22^,^23^,^24^.

Shear flow is also known to induce ordering, crystallization, and phase transitions in surfactant solutions, polymer solutions, and colloidal systems^3^,^14^,^25^,^26^,^27^,^28^,^29^,^30^,^31^,^32^,^33^,^34^,^35^,^36^,^37^,^38^,^39^,^40^,^41^,^42^,^43^,^44^,^45^,^46^,^47^,^48^,^49^,^50^,^51^. The transition from nonionic surfactant micelles to higher order structures (e.g., lamellar, nematic, or onion phases) has been accomplished by coupling shear flow with temperature variations^29^,^30^,^33^,^34^,^35^,^36^,^37^,^38^,^39^,^40^,^41^,^42^. For example, Linemann *et al.*^34^ studied the orientation of C_*m*_E_*n*_ nonionic surfactants in isotropic, cubic and hexagonal phases by using rheo-SALS while varying the temperature from 5 to 45 °C. They observed micellar alignment in both perpendicular and parallel flow directions, caused by the transition from spherical micelles to higher order surfactant structures. Similar structural transitions from isotropic micelles to higher order structures were also observed in ionic surfactant solutions. For example, Berret^39^ reported the formation of shear-induced nematic phases from ionic wormlike micelles under transient shear flows. Rathee *et al.*^29^ studied the crystallization of a lyotropic ionic surfactant solution, proposing that shear-induced modification of spontaneous curvature in amphiphilic molecules could drive phase transition in concentrated bilayers. Similarly, Koppi *et al.*^33^ showed that isotropic diblock copolymers could be ordered under shear, forming lamellar structures perpendicular to the flow-velocity gradient. Safinya *et al.*^35^ reported nematic to Smetic-A phase transition in liquid crystal 4-cyano-4′-octylbiphenyl (8CB) solutions under shear flow, when the temperature was lowered from 40 to 33 °C. For systems with larger characteristic length scales, soft and hard colloidal particles with diameter of ~1 *μ*m have also exhibited abilities to form crystal-like layers when subjected to shear flow, due to delicate balancing among brownian and hydrodynamic forces, and particle-particle interactions^43^,^44^,^45^. Shear flow can reduce the energy barrier for nucleation and accelerate the growth of crystalline phases from a stable isotropic phase, with volume fraction usually ranging *ϕ* ~ 0.2–1.04 and localized Peclet number Pe = *τa*^3^/*k*_*B*_*T* ≥ 1 (*a* is the characteristic length scale in the system, *k*_*B*_*T* is the thermal energy, and *τ* is the shear stress). To summarize, existing literature has shown that proper combinations of raised temperature and shear flow can induce higher order micellar structures from surfactant solutions and other self assembly systems.

In this work, we show that branched micelles and crystal-like structures (CLS) with domain size of tens of nanometers to tens of microns, can be induced from an aqueous nonionic surfactant solution at volume fraction of *ϕ* ~ 0.24, when sheared and strained (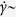 10^3^ s^−1^ and *γ* ~ 10^4^–5 × 10^5^) at moderately low temperature (*T* ≤ 15 °C). We focus on nonionic surfactant system containing polyoxyethylene(20) sorbitan monooleate (Tween-80) and co-surfactant Monolaurin (ML). Tween-80 is an amphipathic, nonionic surfactant composed of fatty acid esters of polyoxyethylene sorbitan, which is frequently used in protein biopharmaceutical formulations and cosmetic industries^6^,^7^. Monolaurin, a lipophilic surfactant, is the most powerful antiviral and antibacterial fatty acid found in coconut oil, which facilitates wormlike micelles formation when used as a co-surfactant^10^,^13^. To our best knowledge, we are the first group reporting the formation of branched micelles and CLS from nonionic Tween-80 spherical micelles under small Pe = 3.5 × 10^−5^ (due to its nanometric micellar diameters), and moderately low temperatures, where high spontaneous curvature of nonionic micelles usually makes it difficult to form higher order structures.

## Results and Discussion

Here, we employed complementary measurement techniques of shear rheometry, rheo-small-angle light scattering (rheo-SALS), birefringence imaging, and cryogenic electron-microscopy (cryo-EM) to capture the structural transition in a nonionic precursor solution containing Tween-80/ML. Tween-80 was purchased from Fisher Scientific (Pittsburgh, PA, USA). ML was a commercial product from Tokyo Chemical Industry Co. Ltd. (TCI AMERICA). All the chemicals were used as received. The precursor is an aqueous solution with 25 wt% of Tween-80 and ML at a weight ratio *χ* = 0.09 (*χ* = ML/(ML + Tween-80)), with *ϕ* ~ 0.24.

### Rheological and Scattering characterizations

**Figure 1:**
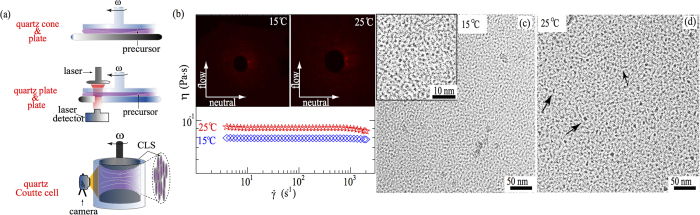
(**a**) Schematics of shear rheology and rheo-SALs setups to obtain the shear rheology and scattering patterns of the precursor at different temperatures and shear rates. (**b**) Shear rheology (steady flow procedure) of the precursor at 15 and 25 °C, exhibiting Newtonian behavior. The insets show representative isotropic scattering patterns in a sheared precursor (~550 s^−1^) at 15 °C and 25 °C. (**c**,**d**) Cryo-EM images of the precursor at 15 °C and 25 °C, displaying mostly spherical micelles at 15 and 25 °C. Some dispersed wormlike micelles were observed at 25 °C (see black arrows in (**d**)).

Quartz cone-plate (50 mm in diameter and 1° truncation angle) geometry from a stress controlled shear rheometer (Anton Paar MCR 502) was used to measure the shear rheology of the precursor. Parallel quartz plate-plate (50 mm of diameter and 5 mm gap) and transparent Couette flow cell (35 mm inner diameter and 1.13 mm gap) with transparent thermo jacket in MCR 502 were used to acquire rheo-optical^52^ features in the precursor solution at 5, 10, 15, and 25 °C (see Fig. 1(a)).

The precursor exhibited Newtonian behavior at 15 and 25 °C, with the zero shear viscosity of 0.07 Pa ⋅ s and 0.09 Pa ⋅ s respectively, implying that the precursor mostly consisted of isotropic spherical micelles at these temperatures (see Fig. 1(b)). Based on the partial ternary phase diagram of water/Tween-80/ML system reported by Sharma *et al.*^10^, both spherical micelles and wormlike micelles can be formed when the concentration of Tween-80 is larger than 20 wt% at 25 °C. Evidenced by the cryo-EM images of the precursor at 15 and 25 °C shown in Fig. 1(c,d), our precursor consists of primarily spherical micelles, along with very few wormlike micelles being observed at 25 °C^10^,^14^.

To verify the structural transition from spherical micelles to branched micelles in the precursor with temperature variations, we conducted time-resolved rheo-small-angle-light-scattering (rheo-SALS) experiments. Figure 1(a) shows the schematics of the rheo-SALS setup of MCR 502, with a 390 *μ*W class 2 He-Ne laser providing monochromatic light at 658 nm wavelength. The laser beam passed through the sample placed between a transparent parallel quartz plate-plate geometry. The light propagated along the velocity gradient direction, thus the scattering patterns represented the precursor structure in the flow-and-neutral plane. The scattering patterns of rheo-SALS are sensitive to orientation fluctuations and concentration gradients of the sample during shear^28^,^36^,^37^,^38^,^52^. We sheared the precursor from 1–600 s^−1^ at 15 and 25 °C, observing isotropic angular distribution of the scattered light intensity, suggesting the presence of isotropic spherical micelles in the precursor^28^,^36^,^37^,^38^. Insets in Fig. 1(b) are representative isotropic scattering patterns of the precursor under shear rate ~550 s^−1^ (see Movie S1). Figure 1(c,d) are cryo-EM images of the precursor at 15 and 25 °C respectively, displaying predominantly spherical micelles (black dots) with a mean diameter of ~9.4 ± 0.3 nm.

**Figure 2:**
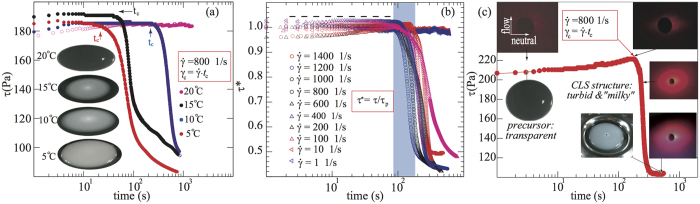
(**a**) Temporal evolution of the shear stress in the precursor at 5, 10, 15, and 20 °C for a fixed shear rate (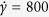 s^−1^). At 20 °C the precursor does not exhibit any distinct structural transition (maroon curve). At lower temperatures (5 and 10 °C as red and black curves) CLS emerged rapidly. The snapshots in (**a**) present the structural transition from isotropic state (at 20 °C) to CLS with “milky” appearance at 15, 10 and 5 °C; arrows in (**a**) correspond to the onset of CLS at a critical time *t*_*c*_, equivalent to a critical strain 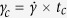. (**b**) Temporal evolution of the normalized shear stress (τ^*^ = *τ*/*τ*_*p*_) under a fixed temperature (15 °C) at varying shear rates. The shear stress was normalized by *τ*_*p*_, the plateau value of shear stress before its decay (see SI). A near universal collapse was observed (blue rectangle), and the onset of the shear stress decay correleated to the CLS formation at critical time *t*_*c*_ ranging from 90–300 s, with total strain in the range of 7.2 × 10^4^–2.4 × 10^5^. (**c**) Temporal evolution of the shear stress at 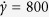 s^−1^ and temperature of 15 °C. The insets show the temporal evolution of the scattering patterns and physical appearance of the precursor. Under shear rate *γ* = 800 s^−1^, when the shearing time is shorter than ~90 s (corresponding strain *γ* < 7.2 × 10^4^), the precursor is transparent with isotropic scattering. When time reaches a critical value *t*_*c*_, the shear stress *τ* of the precursor begins to decay, exhibiting 2-fold anisotropic scattering patterns parallel to the neutral direction. When time goes beyond *t* > 300 s with high strain 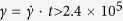, the shear stress abruptly decays and the precursor becomes turbid with a milky appearance, displaying isotropic-like scattering patterns.

**Figure 3:**
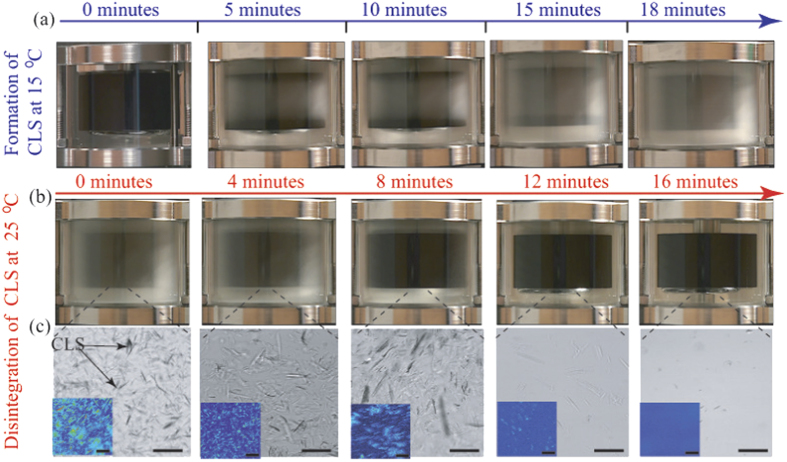
(**a**) Snap shots of the structural evolution in the precursor at 15 °C and 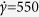 s^−1^, before time reached 18 minutes. Over time, the sheared precursor completely transformed into CLS with “milky” appearance. (**b**) Structural evolution of the CLS once the flow was stopped and the temperature was increased to room temperature. The milky color and consistency of the CLS gradually disappeared over time. The CLS completely disintegrated after ~16 minutes, with the precursor reversed to its original transparent appearance. See SI movies. (**c**) Microstructure of the micro-crystals formed in the precursor gradually disintegrated over time at room temperature. The insets are birefringence images of the CLS at equilibrium. All scale bars represent 10 *μ*m.

**Figure 4:**
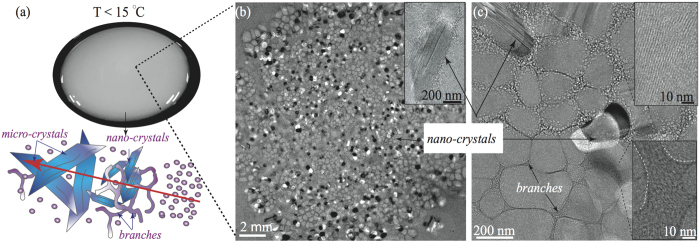
(**a**) The CLS exhibits turbid and “milky” appearance. The cartoon represents the transition from spherical micelles to CLS. Different sizes of needle-like blue objects represent either nanocrystal-like or microcrystal-like entities. (**b**) Low magnification cryo-EM image of the CLS: showing wormlike micelles, branched networks, and nano-crystals (white-black domains and inset). (**c**) High magnification cryo-EM images of the CLS, exhibiting wormlike micelles and branched networks. CLS consists of branched micellar bundles, formed from individual wormlike micelles. The insets display the stacking of individual wormlike micelles. Wormlike micelles in the CLS have a diameter of ~9.4 ± 0.3 nm, while the micellar branches diameter ranges from tens to hundreds of nanometers.

When the precursor solution was subjected to a constant shear rate with sufficient amount of time, the isotropic spherical micelles in the precursor evolved into CLS (confirmed by a combination of rheo-SALS, cryo-EM and birefringence images, shown in Figs 2, 3, 4 and Figures S2–S5, more discussions to be followed), with turbid and “milky” consistency at temperatures ranging from 5–15 °C. Figure 2(a) shows the temporal evolution of the shear stress *τ* under a fixed shear rate (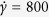 s^−1^), at temperatures of 20, 15, 10, and 5 °C. At 20 °C (maroon circles in Fig. 2(a)), *τ* remained constant beyond 1000 s, suggesting that microstructures in the precursor remained isotropic and the precursor persisted to be transparent (see inset pictures in Fig. 2(a)). At 15 °C, *τ* remained constant before reaching a critical time *t*_*c*_, where the critical strain can be approximated by 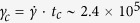. Beyond *γ*_*c*_, the shear stress of the precursor abruptly decayed, suggesting spherical micelles to CLS transition in the precursor (blue circles in Fig. 2(a)). The critical strain *γ*_*c*_ required for the onset of the shear stress decay decreased by decreasing temperatures (see black circles of 10 °C and red circles of 5 °C, shown in Fig. 2(a)), indicating that the activation energy required to form CLS is reduced at lower temperatures. Figure 2(b) shows the normalized shear stress τ^*^ = *τ*/*τ*_*p*_ plotted against time, at varying shear rates under fixed temperature of 15 °C, with *τ*_*p*_ the plateau value of *τ* before decay (the black dashed line in Fig. 2(b) depicts the plateau region of *τ*), see more details in Figure S1. For 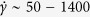 s^−1^, the total strain (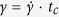) required to induce spherical micelles-to-CLS transition was on the order of 10^4^–10^6^. Moreover, the precursor did not form CLS (within the experimental time studied) when subjected to shear rates between 1–10 s^−1^ (flat curves in Fig. 2(b)). High shear rates (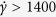 s^−1^) were not examined due to inertia effects in the rheometer.

We further verified the structural transition from spherical micelles to CLS by examing the scattering patterns obtained from rheo-SALS, focusing on the transition temperature (15 °C) where CLS began to emerge. Figure 2(c) displays a representative rheo-SALS plot of shear stress *τ* versus time for the precursor at fixed temperature of 15 °C and fixed shear rate of 800 s^−1^. At time *t* < 90 s (corresponding strain 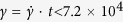), the precursor remained transparent with isotropic scattering. The shear stress slightly increased from 205 Pa to 230 Pa when the precursor was sheared less than 200 s, corresponding to the onset of the structural transition from spherical micelles to CLS, evidenced by the turbid color in the precusor, further accompanied by anisotropic 2-fold scattering patterns parallel to the neutral direction from the rheo-SALS measurements. When time *t* reached a critical value *t*_*c*_ ~ 200 s, equivalent to 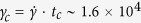, the shear stress *τ* of the precursor begins to decay, with the precursor exhibiting 2-fold anisotropic scattering patterns parallel to the neutral direction, see Fig. 2(c). When time goes beyond *t* > 300 s with high strain 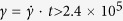, the shear stress abruptly decayed and the scattering pattern of the CLS evolved from 2-fold pattern into isotropic scattering pattern, implying that most spherical micelles present in the precursor transformed into CLS (see Movie S2). At this stage the precursor became completely turbid with “milky” consistency (see inset image in Fig. 2(c)). We speculate that due to the coupling between low temperature and high strain (*T* < 15 °C and *γ* ~ 10^4^–10^6^), the spherical micelles in the precursor are initially aligned in the flow direction, subsequently stacked in both neutral and velocity gradient directions over time, inducing micellar growth, creating possible nucleation sites for crystallization^29^,^30^,^33^,^34^,^35^,^36^,^37^,^38^,^39^,^40^,^41^,^42^,^43^,^44^,^46^,^47^. This observation is consistent with existing reports for both surfactants and colloidal solutions^29^,^30^,^33^,^34^,^35^,^36^,^37^,^38^,^39^,^40^,^41^,^42^,^43^,^44^,^46^,^47^, where isotropic phases could grow in the neutral direction of the flow velocity.

We also confirmed the existence of CLS in the precursor by directly visualizing the sheared precursor in a transparent temperature-controlled Couette flow cell attached to MCR 502, combined with a birefringence imaging microscope (EXICOR^®^ MICROIMAGER^TM^, Hinds Instruments, Inc.). After loading the precursor in the Couette cell at room temperature, the temperature was decreased from 25 °C to 15 °C at 3 °C/minute, equilibrated for 20 minutes before each experiment, see schematics in Fig. 1(a). Figure 3(a) shows snap-shots of the structural evolution of the precursor at 15 °C, up to total strain of 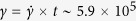. The precursor did not exhibit any structural transformation initially. However, CLS began to emerge beyond five minutes, eventually the entire sample became highly turbid with “milky” appearance (see Movie S3 and Figure S2). When the imposed shear was removed, the CLS in the precursor remained stable as long as the temperature was maintained at ≤15 °C. The CLS in the precursor disintegrated within 16 ± 2 minutes once the temperature increased to ~23 °C, see Fig. 3(b,c) and Movie S4. We also extracted precursor from the Couette cell at each experimental condition and imaged the precursor using a birefringence imaging microscope, which is based on the PhotoElastic Modulator technology, with a digital resolution of 0.01 nm, a detection limit of 0.1 nm and a measurement range of 300 nm and above (red light). The CLS in the precursor at 15 °C exhibited crystal-like structures with an average domain size of 20 *μ*m (see Fig. 3(c) and Figure S3). The birefringence response in the precursor disappeared at room temperature, implying that anisotropic CLS reversed back to isotropic spherical micelles in the solution (see insets in Fig. 3(c)). The connection points in branched micelles (see next section) can slide against each other, promoting high fluidity, and therefore reducing the shear viscosity in the precursor^16^,^17^,^18^,^19^,^20^,^21^,^22^,^23^,^24^. Moreover, crystal-like objects observed in the precursor can potentially slide against each other when subjected to shear flow, reducing the shear viscosity in the precursor even further^45^,^51^. Similar rheological behavior was also observed at 5 and 10 °C, displaying Newtonian behavior with shear viscosity of ~0.05–0.09 Pa ⋅ s.

### Microstructural analysis

We conducted cryogenic electron-microscopy (Cryo-EM) to analyze the microstructure of the precursor at different temperatures. We used a Vitrobot (FEI) to prepare the samples at 10, 15 and 25 °C with controlled 100% of humidity during sample preparation. At 10–25 °C the precursor consists of spherical micelles with a diameter of ~9.4 ± 0.3 nm (Fig. 1(b)). The cartoon in Fig. 4(a) illustrates the structural transition from spherical micelles to CLS, with nanocrystal-like structures being embedded in the micellar branches, while microcrystal-like structures possibly nucleated from branched micelle aggregates and nano-crystal nuclei. The CLS has milky color (Fig. 4(a)), showing highly branched networks with embedded nanocrystals (white and black spots in Fig. 4(b) and inset in (b)), exhibiting characteristic domains of tens of nanometers (see Figures S3–S5). Nanocrystal-like domain with size range of 50–500 nm is embedded in the branched micellar networks (see Fig. 4(c)), with microcrystal-like domain with size range of 1–30 *μ*m floating in the CLS solution (see Figure S3 in SI). Figure 4(b,c) illustrate low and high magnification cryo-EM images of nanocrystal-like structures (black and white spots) being embedded in the branched micellar networks.

**Figure 5:**
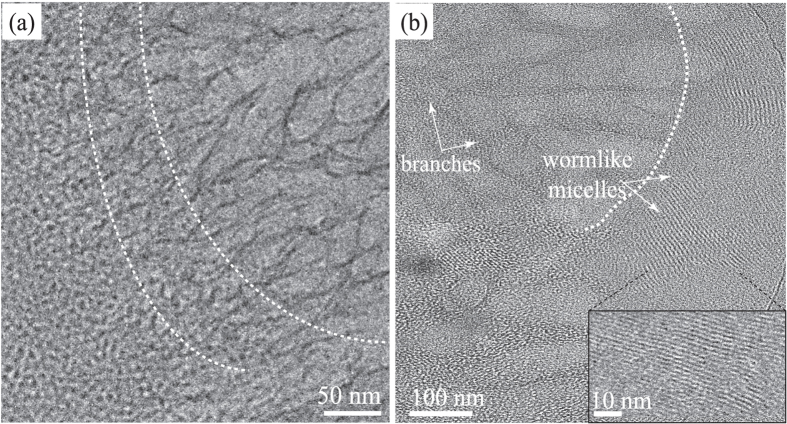
(**a**) High magnification cryo-EM image captures instantaneous structural transition in the precursor from spherical micelles to wormlike micelles, with subsequent transition into branched structures at 15 °C. (**b**) High magnification cryo-EM image seizes the transition from wormlike micelles to branched micellar networks in the precursor, highlighted by white dashed lines.

Figure 5 are high magnification cryo-EM images showing the structural transition from individual wormlike micelles (with diameter of ~9.4 ± 0.3 nm) to branched networks with “Y-fold” junctions, with dimensions reaching as large as 500 nm with branch diameters ranging from 10–100 nm. Branches in the CLS are composed by micellar bundles, see inset in Fig. 5(b)^14^. Figure 5(a,b) captured instantaneous structural transition in the precursor from spherical micelles to wormlike micelles, with subsequent transition into branched structures. Before proceed, we would like to emphasize that cryo-EM sample preparation procedure can inevitably induce shear rates on the order of 10^3^–10^5^ s^−1^: the velocity on the blotting paper during cryo-EM sample preparation lies around few cm ⋅ s^−1^, with the thickness of the sample in tens to hundreds of nanometers. It has been reported that such high shear rates might cause microstructural modifications in micellar solutions^40^,^41^,^42^. To our advantage, high shear rate (~65,000 s^−1^) was introduced in the precursor during the sample preparation, spontaneously inducing branched micelles and CLS formation. In addition, we observed long wormlike micelles and CLS in the precursor prepared at 10 °C, indicating that Tween-80 spherical micelles are able to form elongated wormlike micelles at low temperatures where the spontaneous curvature is expected to be high (see Figure S4 in SI). We also examined the thermo-reversibility of the precursor containing CLS. The CLS completely disintegrated at room temperature where spherical micelles re-appearead and the precursor recovered its translucent color (see Fig. 1(c,d)). Moreover, when the transparent precursor was re-strained (*γ* > 10^4^) at ≤ 15 °C, the CLS re-emerged (see Fig. 3 and Figure S2).

**Figure 6:**
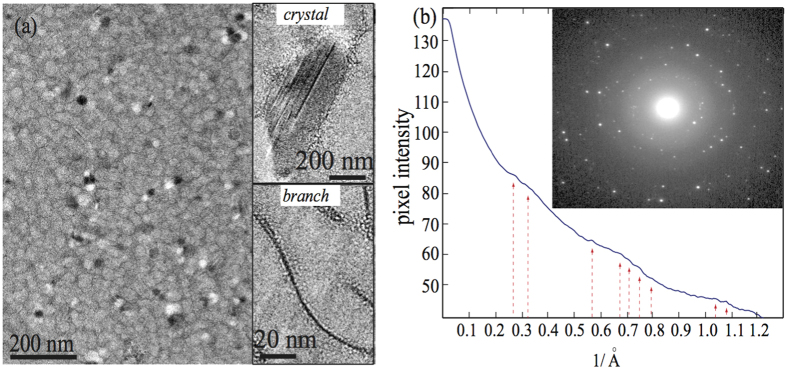
(**a**) Representative cryo-EM image of the CLS. The white and black spots are nanocrystal-like domains in the CLS. The insets display the crystal-like structures and micellar branches in the CLS. (**b**) Rotationally averaged radial intensity obtained from the CLS. The inset shows the diffraction patterns of the CLS, exhibiting some symmetry and lattice order in the CLS.

To further examine CLS in the precursor, we analyzed characteristic diffraction patterns of cryo-EM images of multiple CLS samples (see Fig. 6). In most transmission electron microscopes, there is a net rotation between an image and its corresponding diffraction pattern. We followed the work by Mitchell^53^ to obtain the rotational averaged intensity of the diffraction patterns of the CLS by using the software DigitalMicrograph^*TM*^ and its scripts (DigitalMicrograph Scripting, DiffTools), to estimate the diffraction peaks of our CLS samples. Figure 6(b) shows all peak locations in the rotationally averaged radial intensity profile of the CLS. The inset in Fig. 6(b) displays a representative image of the diffraction patterns of the CLS, alluding some symmetry with a reasonably internal lattice order in the CLS (see more details in Figure S5).

**Figure 7:**
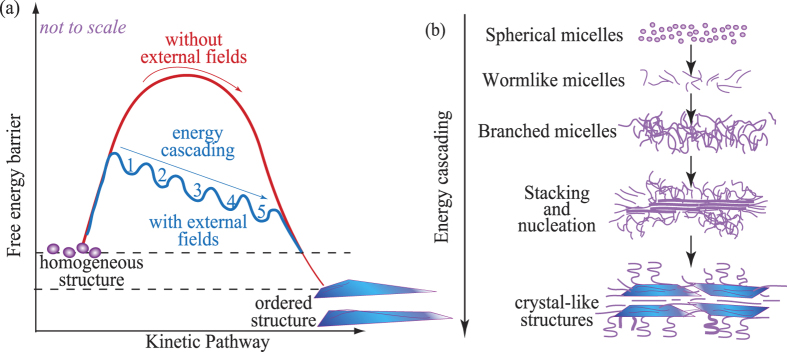
(**a**) Schematic representation of the flow-induced cascading reduction of a free energy barrier. Initially the system possesses homogeneous structures (i.e., spherical micelles in purple dots). External fields (e.g., coupling shear flow and low temperature in our case) suppress the excessively large energy barrier to form ordered structures by triggering the formation of dissipative structures (i.e., crystal-like structures) depicted in multiple states (blue curve). In the absence of external fields, homogenous structures require significantly higher amount of energy to form ordered structures (red curve). (**b**) Under external fields, spherical micelles can follow the free energy landscape trajectory (blue curve shown in (**a**)) and form dissipative structures such as wormlike micelles, branched micelles, triggering the formation of unfolded/aligned/stacked branched micelle aggregates and nano-crystal nuclei, eventually transforming into crystal-like structures (CLS)^47^.

Summarizing our findings, we suggest that transition from spherical micelles to CLS at *T* ≤ 15 °C, 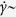 10^3^ s^−1^, and *γ* ~  10^4^–10^6^ is likely to undergo a similar energy cascading mechanism as those observed in non-equilibrium binary systems (i.e., flexible polymers)^46^,^47^, see cartoons in Figs 4(a) and 7. The self-organization of molecular assemblies in non-equilibrium binary solutions depends on the competition between the intrinsic rate of the system (Γ), and the rate introduced by the external field^47^. In our case, the external rate corresponds to the shear rate 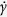 when the nonionic precursor is sheared at 15 °C, while the intrinsic rate could be the relaxation rate of the local concentration fluctuations of nonionic micelles. If 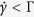, the entropic fluctuations quickly dissipate under shear flow, hence the microstructure of the precursor remains unaltered. However, if 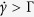, entropic fluctuations are influenced by the shear flow and temperature, triggering a flow-induced cascading reduction in the free energy barrier, promoting spherical micelles to follow the free energy landscape trajectory (blue curve shown in Fig. 7(a)) and form dissipative structures^46^,^47^. Based on the measurements from rheo-SALS, birefringence imaging, and cryo-EM, we suggest that the nonionic spherical micelles would first form micellar strings under shear, which fuse and develop into elongated wormlike micelles^48^,^49^,^50^. Secondly, wormlike micelles become longer and entangled, inducing branched network formation. Branched micelles in the precursor can further unfold, align, and stack along the flow direction, forming nano-crystal nuclei, eventually transforming into crystal-like structures (CLS), see Fig. 7(b).

Creating higher order nano- and micro-structures from nonionic surfactants is desirable due to their electrolyte-free environment, biocompatibility, and less eco-toxic properties. Our results provide a simple alternative to form crystal-like structures from nanometric nonionic spherical micelles for encapsulation, purification, and crystallization applications that are sensitive to ionic environments and high temperatures^11^,^54^. For example, identifying crystallization and purification conditions for new proteins is challenging and time consuming^54^. One common practice in protein crystallization is to expose proteins at different concentrations under a variety of external conditions (i.e., precipitant types with varying concentrations, pH, and temperatures) to induce crystalline precipitation, phase separation, and crystal formation^54^. Our work demonstrates that the coupling between temperature and shear flow can be used as an effective flow modulator to induce protein crystallization without changing the protein concentration and/or pH.

## Conclusions

We described the evolution of the microstructure and rheological properties of a biocompatible nonionic surfactant solution, under proper combinations of temperature and flow conditions. Structural transition can be induced from spherical micelles to branched micelles and CLS in a Tween-80 based precursor solution. The precursor exhibited isotropic scattering patterns at 15 and 25 °C, implying the presence of spherical micelles. Spherical micelles evolved into CLS when coupling low temperature (≤15 °C) with high shear rate (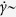 10^3^ s^−1^) and high strain (*γ* ~ 10^4^–10^6^). By keeping the temperature ≤ 15 °C, the CLS remained stable even after the cessation of the shear flow. However, at ~23 °C the CLS completely disintegrated after ~16 minutes. Cryo-EM techniques provided further evidence of branched micelles and CLS morphologies in the precursor. Two domains in the cryo-EM samples were observed in all samples analyzed: first domain exhibited spherical micelles in the cryo-EM samples, while the second domain displayed either branched micelles or CLS, consistent with our rheo-SALS and shear rheometry measurements. The high shear rate and high strain during shear flow overpass the energy penalties imposed by the high curvature energy of spherical micelles to form elongated micelles, “Y-fold junctions”, branched networks, and CLS. To the best of our knowledge, we are the first group illustrating high resolution images of wormlike micelles, branched micelles, and CLS structures created from Tween-80 based nonionic surfactant solutions, when sheared at relatively low temperatures.

## Methods

### Precursor preparation

Polyoxyethylene(20) sorbitan monooleate, also known as Tween-80, was purchased from Fisher Scientific (Pittsburgh, PA, USA). Monolaurin (ML) was a commercial product from Tokyo Chemical Industry Co. Ltd., America (TCI AMERICA). All the chemicals were used as received. Our precursor solution was an aqueous solution with 25 wt% of Tween-80 and ML at a weight ratio *χ* = 0.09 (*χ* = ML/(ML + Tween-80)). At this concentration the precursor predominantly consists of spherical micelles, while coexisting with rather few wormlike micelles at room temperature^10^,^14^. The precursor was prepared by adding appropriate amount of Tween-80 and ML in deionized (DI) water and mixing at room temperature for 1 day, and then leaving the solution at rest for 2 days to ensure equilibrium before experiments.

### Rheometry and rheo-SALS

We used the small-angle-light-scattering (SALS) system in a stress controlled shear rheometer (Anton Paar MCR 502) to investigate shear-dependent microstructure changes of our micellar system during bulk shear rheological measurements. The temperature was varied from 15 to 23 °C by using a solvent trap and thermo jacket. A quartz cone-plate geometry (50 mm in diameter and 1 ° of truncation angle), plate-plate (50 mm in diameter and gap size of 0.2 mm), and transparent Couette flow cell (30 mm inned diameter and 27 mm hight) were used for all measurements. A rheo-SALS (Anton Paar) with 390 *μ*W class 2 He-Ne laser provided monochromatic light at wavelength 658 nm. The laser beam was deflected and passed through the sample placed between the transparent quartz plate-plate geometry. The scattering images formed on the screen were captured using a monochrome camera (Rheoplus Camera Anton Paar).

### Birefringence imaging

We used a birefringence imaging microscope (EXICOR^®^ MICROIMAGER^TM^, Hinds Instruments, Inc.) to observe the orientation of micellar structures during flow, based on PhotoElastic Modulator (PEM) technology, with a digital resolution of 0.01 nm, a detection limit (noise floor) of 0.1 nm and a measurement range of beyond 300 nm (using red light).

### Cryo-EM

The freeze-plunging method was conducted with Vitrobot Mark IV (FEI, USA). 1.5 *μ*L of the sample was applied on washed and glow-discharged Quantifoil R2/1 copper grids (Quantifoil Micro Tools, Germany). Blotting excess solution was done twice for 4 seconds under 100% humidity for each sample (10, 15, and 25 °C). The sample was then immediately plunge frozen by immersing into a reservoir with liquid ethane cooled by liquid nitrogen. The grids with the frozen sample were transferred under liquid nitrogen to Titan Krios Autoloader (FEI, USA) while keeping the temperature below −160 °C. Cryo-EM imaging was performed at 300 kV at stage temperature of −193 °C. Electron microscopy images were recorded with a 4k × 4k direct electron detector, Falcon II camera (FEI, USA) at low dose mode.

## Additional Information

**How to cite this article**: Cardiel, J. J. *et al.* Formation of crystal-like structures and branched networks from nonionic spherical micelles. *Sci. Rep.* **5**, 17941; doi: 10.1038/srep17941 (2015).

## Supplementary Material

Supplementary Information

Supplementary Video S1

Supplementary Video S2

Supplementary Video S3

Supplementary Video S4
