# Supplementary material for: Formation of crystal-like structures and branched networks from nonionic spherical micelles
Source: Sci Rep. 2015 Dec 9;5:17941. doi: 10.1038/srep17941 (PMC4673692; doi:10.1038/srep17941)
Supplement: Supplementary Information [file srep17941-s1.pdf]

Formation of crystal-like structures and branched networks from nonionic spherical micelles

Joshua J. Cardiel[1], Hirotoshi Furusho[2], Ulf Skoglund[2], Amy Q. Shen[1*]

[1] Micro/Bio/Nanofluidics Unit, Okinawa Institute of Science and Technology Graduate University, Okinawa, Japan

[2] Structural Cellular Biology Unit, Okinawa Institute of Science and Technology Graduate University, Okinawa, Japan

[*] amy.shen@oist.jp

1 Supplementary Figures

Additional Rheological and Rheo-SALS Characterizations

We investigated the temporal evolution of the shear stress τ in the precursor at a fixed temperature of 15 °C, with varying shear rates ($\dot{\gamma}$=1–1400 s^{-1}), see Figure 1. For shear rates $\dot{\gamma} \leq 200$ s^{-1}, the shear stress τ maintained as a constant over time, indicating that the precursor solution remained isotropic. For shear rates ranging between 200–1400 s^{-1}, the shear stress underwent a plateau region when reaching a critical strain $\gamma_c = \dot{\gamma} \times t_c \sim 10^4$–$10^6$, see blue rectangular region in Figure 1. Beyond γ_c, the shear stress of the precursor abruptly decayed, implying

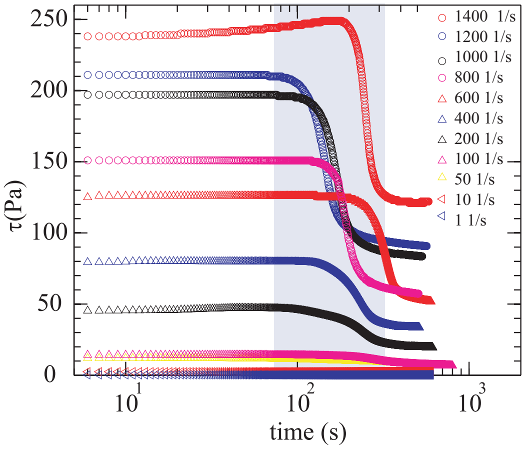

Fig. S1: Temporal evolution of the shear stress τ for a fixed temperature (15 °C), at varying shear rates.

microstructural transition from isotropic spherical micelles to crystal-like structures (CLS) in the precursor, as confirmed by a combination of rheo-SALS, cryo-EM and birefringence images (see Figures S2–4 in this SI document and additional images in the main manuscript).

In order to qualitatively visualize the structural transition in the precursor when sheared, we loaded the precursor into a transparent temperature controlled Couette flow cell. The temperature was decreased from 25 °C to 15 °C at 3 °C/minute, equilibrated for 20 minutes before shearing the precursor, see schematics in Figure S2 (a). We varied the shear rate $\dot{\gamma}$ from 1 to 1000 s^{-1}, observing microstructural transition at $\dot{\gamma} \geq 200$ s^{-1} and a total strain

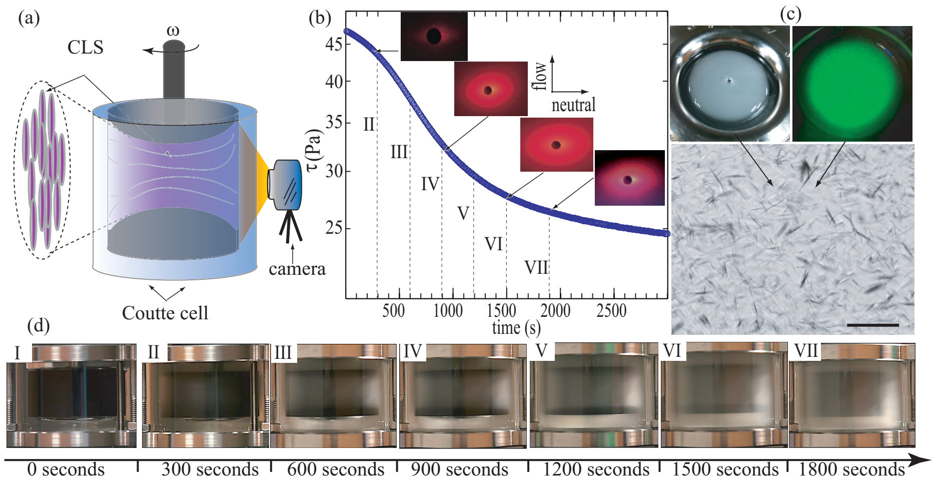

Fig. S2: (a) Schematics of the transparent Couette cell used to visualize the structural transition of the precursor. (b) Temporal evolution of shear stress τ with fixed shear rate $\dot{\gamma}$=550 s^{-1} and temperature of 15 °C. The inset images show the temporal evolution of the scattering patterns from isotropic to anistropic structures. (c) Snap shots of the precursor at 15 °C extracted from the Couette cell, after being sheared at $\dot{\gamma}$=550 s^{-1} with a total strain of $\gamma \sim 1.9\times10^4$. Left image is under white light while right image is under polarized light. Scale bar is 10 μm. (d) At $\dot{\gamma}$=550 s^{-1} and temperature of 15 °C, sheared precursor transformed from transparent to milky appearance over time.

of $\gamma \sim 10^4$–10^6.

Figure S2 (b) shows the evolution of the transient shear stress and corresponding scattering patterns in the precursor over time. The shear stress decayed beyond the onset formation of the CLS in the precursor, indicating that the CLS rich precursor posseses high fluidity due to the presence of branched micelles and slip crystals in the CLS [2, 3]. Figure S2 (c) shows the microstructure and physical appearance of the CLS. Figure S2 (d) shows snap shots of the precursor inside the Couette cell at 15 °C when sheared at $\dot{\gamma}$=550 s^{-1}. From zero seconds to ∼300 seconds the precursor remained transparent (visible with our setup). The precursor started to exhibit anisotropic scattering patterns beyond 300 seconds, and eventually the CLS-rich precusor exhibited milky appearance (see Figure S2 (b & d (I–VII))).

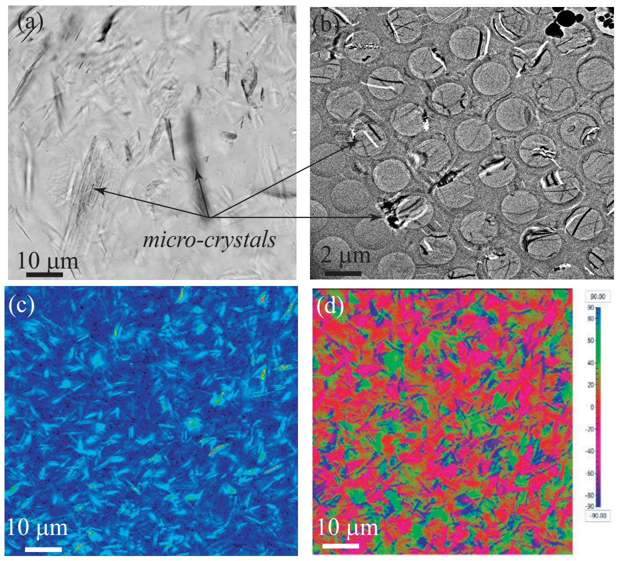

Fig. S3: (a) Microcrystal-like structure in the precursor captured by an inverted microscope (40×). (b) Low magnification cryo-EM image of microcrystal-like structures shown as white and black objects. (c&d) Birefringence images of the CLS-rich precusor; (c) shows the retardation behavior and (d) presents the angular distribution of microcrystal-like structures in the precursor.

Cryo-EM and microscopy images

For microcrystal-like structures with size range of 1-30 μm floating in the precursor, they are illustrated by a standard inverted optical microscope (Figure S3 (a) with 40×) and a birefrengence microscope (Figure S3 (c & d)). These microcrystal-like structures have high aspect ratio (length/width ∼500±150), indicating anistropy in the precursor. Figure S3 (c &d)) display both birefrin-

gence and angular orientation in the precursor. We suggest that once the precursor cotaining CLS is sheared, both nanocrystal-like and microcrystal-like structures can be oriented in the flow direction, displaying isotropic scattering patterns as those observed in our rheo-SALS studies (see Figure 2 and Figure 3 in the main manuscript). Microcrystal-like structures can also be observed from the low magnification cryo-EM image, see arrows pointing to the white and black domains in Figure S3 (b). The cicular patterns in Figure S3 (b) are part of the cryo-EM grid.

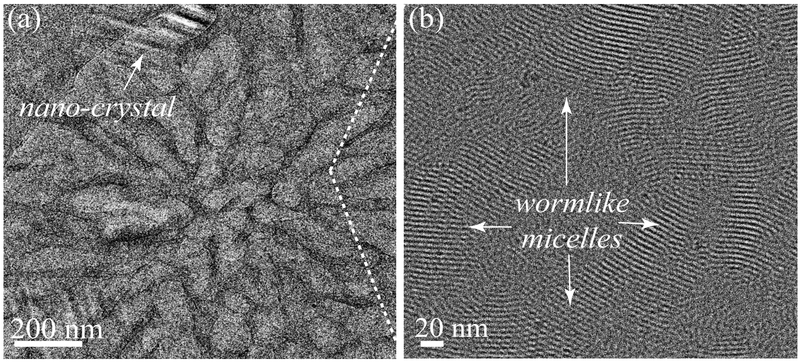

Fig. S4: (a&b) cryo-EM images of branched micelles and wormlike micelles formed at 10 °C.

We also observed that Tween-80 nonionic precursor solution possesses the ability to form long wormlike micelles and branched micelles at 10 °C, indicating that Tween-80 spherical micelles are able to form elongated wormlike micelles at low temperatures where the spontaneous curvature is expected to be high (see Figure S4).

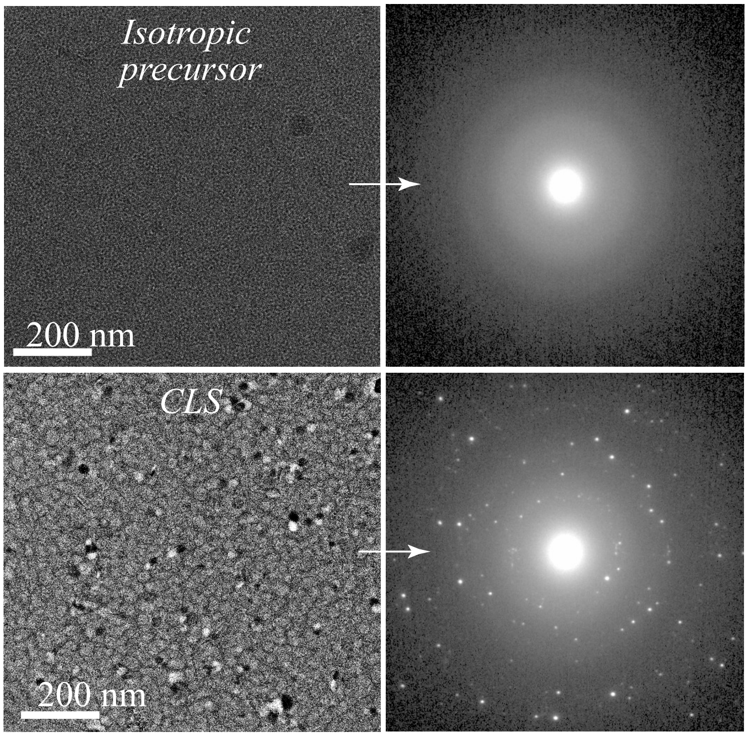

Fig. S5: cryo-EM images with their corresponding diffraction patterns of the isotropic precursor (top panel) and CLS structure (bottom panel).

To further examine CLS in the precursor, we analyzed characteristic diffraction patterns from cryo-EM images in multiple CLS samples (see details of methods in the main manuscript). The top panel in Figure S5 shows a

representative cryo-EM image of the precursor with spherical micelles (∼9.4 nm of diameter), and its corresponding isotropic diffraction patterns. Whereas the bottom panel shows a representative cryo-EM image of CLS-rich precursor, displaying branched micelles with embedded crystal-like structures (white and black spots). The diffraction patterns exhibit some symmetry and internal lattice order, which are characteristic of crystal-like structures.

2 Supplementary Videos

- SI video 1: Rheo-SALS for the precursor at shear rate of 550 1/s, at 15 °C, by using a plate-plate geometry in MCR 502 rheometer.
- SI video 2: Rheo-SALS for the precursor at shear rate of 800 1/s, at 15 °C, by using a plate-plate geometry in MCR 502 rheometer. The 3 movie clips highlight the isotropic to anisotropic scattering transition.
- SI video 3: Transparent Couette cell was used to visualize the formation of the crystal-like structure in the precursor at 15 °C.
- SI video 4: Transparent Couette cell was used to visualize the disintegration of the crystal-like structure in the precursor at 23 °C.

References

[1] Dan N and Safran S A (2006) *Junctions and End-Caps in Self-assembled Non-ionic Cylindrical Micelles.* Adv. Coll. and Interf. Sci. 123-126:323-331.

[2] Hess S (1985) *Shear-Induced Melting and Reentrant Positional Ordering in a System of Spherical Particles.* Int. J. Thermophys. 6:657–671

[3] Lopez-Barron L, Wagner N J and Porcar L (2015) *Layering, melting, and recrystallization of a close-packed micellar crystal under steady and large-amplitude oscillatory shear flows.* J. Rheol. 59:793-820.
